# An *in situ* USAXS–SAXS–WAXS study of precipitate size distribution evolution in a model Ni-based alloy^1^

**DOI:** 10.1107/S1600576717006446

**Published:** 2017-05-30

**Authors:** Ross N. Andrews, Joseph Serio, Govindarajan Muralidharan, Jan Ilavsky

**Affiliations:** a Argonne National Laboratory, USA; b Oak Ridge National Laboratory, USA

**Keywords:** ultra-small-angle X-ray scattering, small-angle X-ray scattering, wide-angle X-ray scattering, USAXS–SAXS–WAXS, precipitation hardening, Bayesian inverse transformation

## Abstract

Intermetallic γ′ precipitates typically strengthen nickel-based superalloys. The shape, size and spatial distribution of strengthening precipitates critically influence alloy strength, while their temporal evolution characteristics determine the high-temperature alloy stability. Combined ultra-small-, small- and wide-angle X-ray scattering (USAXS–SAXS–WAXS) analysis can be used to evaluate the temporal evolution of an alloy’s precipitate size distribution (PSD) and phase structure during *in situ * heat treatment. Analysis of PSDs from USAXS–SAXS data employs either least-squares fitting of a preordained PSD model or a maximum entropy (MaxEnt) approach, the latter avoiding *a priori* definition of a functional form of the PSD. However, strong low-*q* scattering from grain boundaries and/or structure factor effects inhibit MaxEnt analysis of typical alloys. This work describes the extension of Bayesian–MaxEnt analysis methods to data exhibiting structure factor effects and low-*q* power law slopes and demonstrates their use in an *in situ* study of precipitate size evolution during heat treatment of a model Ni–Al–Si alloy.

## Abstract 2

Combined ultra-small-, small- and wide-angle X-ray scattering (USAXS–SAXS–WAXS) provides *in situ* evaluation of the precipitate size distribution (PSD) and phase structure temporal evolution during heat treatment. A method for extraction of an arbitrary PSD in the presence of interparticle interactions is described and illustrated for study of PSD evolution.

## 1. Introduction   

The formation of fine, coherent, ordered, intermetallic 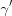 precipitates in a face-centered cubic matrix strengthens Ni-based superalloys (Sims *et al.*, 1987 ▸; Reed, 2008 ▸). The design and development of precipitation-strengthened alloys requires an understanding of the kinetics of precipitate nucleation, growth and Ostwald ripening (coarsening). The shape, particle size distribution (PSD) and spatial distribution of precipitates critically influence alloy strength. Thus, many studies have addressed the kinetics of 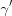 growth and coarsening (Sims *et al.*, 1987 ▸; Reed, 2008 ▸; Jayanth & Nash, 1989 ▸, 1990 ▸; Ardell, 1999 ▸; Baldan, 2002*a* ▸,*b* ▸). In addition, coarsening kinetics dictate the long-term structural stability of alloys. Various factors, including phase equilibria, diffusion kinetics, elastic properties and precipitate structure, influence temporal and spatial precipitate evolution during coarsening. Continued interest in the prediction of coarsening behavior, particularly in complex engineering alloys, has inspired correlation of *in silico* PSD evolution simulation results with *in situ* studies (Jayanth & Nash, 1989 ▸, 1990 ▸; Ardell, 1999 ▸; Baldan, 2002*a* ▸,*b* ▸; Voorhees, 1985 ▸, 1992 ▸).

Classical methods for determination of PSD temporal evolution involve *ex situ* aging and microscopy on a series of different samples aged for different times. *In situ* X-ray or neutron scattering provides an alternative method for PSD study using sequential measurements on a single sample undergoing heat treatment. In particular, combined ultra-small-, small- and wide-angle X-ray scattering (USAXS–SAXS–WAXS) allows the study of not only precipitate growth and coarsening by evaluation of the PSD (from USAXS–SAXS) temporal evolution, but also the phase structure (from WAXS) as a function of heating time. Evaluation of the scattering intensity 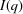 obtained from USAXS–SAXS in terms of a PSD generally employs either a functionally defined PSD or a maximum entropy approach.

Lifshitz & Slyozov (1961 ▸) and Wagner (1961 ▸) developed a theory (LSW theory) describing coarsening behavior of a vanishingly small volume fraction of precipitates within a matrix, predicting scaling of the average radius 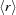 with time *t* as 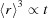 and a time-invariant PSD. LSW theory further specifies a functional form for the PSD 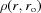 characterized by a size parameter 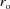 to yield a distribution of particle radii *r*: 
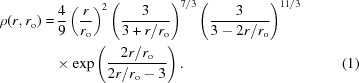
Assuming a spherical precipitate geometry, integration of the LSW distribution 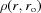 over a spherical form factor 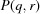 gives the expected scattering intensity 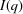 from an LSW distribution of spherical precipitates: 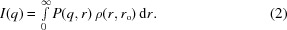
However, often a broader-than-LSW PSD occurs in real systems, which other theories address by development of alternative models for the PSD. For example, Ardell & Ozolins (2005 ▸) developed the TIDC model of precipitation hardening, defining a PSD that incorporates a shape parameter in addition to a characteristic radius 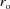.

Unfortunately, *a priori* definition of a defined PSD shape for small-angle scattering (SAS) data analysis can give misleading results. The knowledge imparted by such a distribution ordinarily exceeds the information available from the scattering experiment – the simple fact that a model fits the data does not, by itself, make it the most appropriate model. Rather than beginning with a defined functional form for the PSD, inverse transform approaches seek an arbitrary size distribution 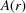 that explains the observation 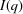:
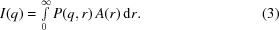
Truncation of the data, the presence of noise, slit integration limits and resolution effects complicate determination of 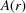 given only the data 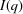, as many different solutions fit the measured data within the experimental uncertainty. Selection of the most likely solution from this set relies on the principle of parsimony, which says to select the simplest PSD sufficient to explain the SAXS observation.

Various techniques exist for determination of the most likely inverse transform solution from SAS data, including the point of inflection (Glatter, 1977 ▸), perceptual criteria (Svergun, 1992 ▸) and maximum entropy (MaxEnt) (Jemian & Allen, 1994 ▸; Hansen & Muller, 1996 ▸). MaxEnt methods have the advantage that 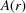 does not depend upon subjective decisions about the solution; the data and experimental uncertainty in conjunction with a functional prior expectation 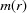 alone dictate the solution 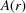. MaxEnt finds a solution by minimizing 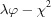 (rather than 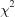 alone), where φ characterizes the deviation of the solution from a user-supplied prior expectation: 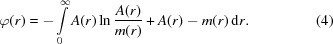
The Lagrange multiplier λ controls the relative weight of misfit 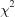 and prior functional φ. External knowledge about the form of the PSD could suggest a particular form (such as the LSW distribution) for the prior expectation function 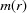, in which case MaxEnt and conventional fitting would be likely to give similar results. However, often X-ray scattering analysis provides the only insight into PSD evolution (*e.g.* in the absence of costly *ex situ* transmission electron microscopy studies), in which case MaxEnt methods should employ the least informative prior expectation possible, a flat 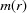.

An uninformative flat prior for 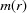 conveys no information about the form of the distribution; expecting a flat PSD at the outset means that new information (peaks) in 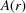 comes only from the measurement. Taylor expansion of equation (4) with a flat prior estimate function 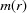 reveals equivalence between the entropy functional φ and traditional smoothness regularization (Hansen, 2000 ▸); the assumption of a flat prior corresponds to the usual smoothness (sum of second derivative) criteria. Bayesian techniques developed and implemented in the program *IFTc* (Hansen, 2012 ▸, 2014 ▸) enhance the traditional MaxEnt framework by finding the most likely value for λ without employing the traditional relation between 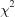 and the number of data points or relying on an *ad hoc* selection of λ. Instead, the data and measurement error inherently dictate λ.

**Figure 1:**
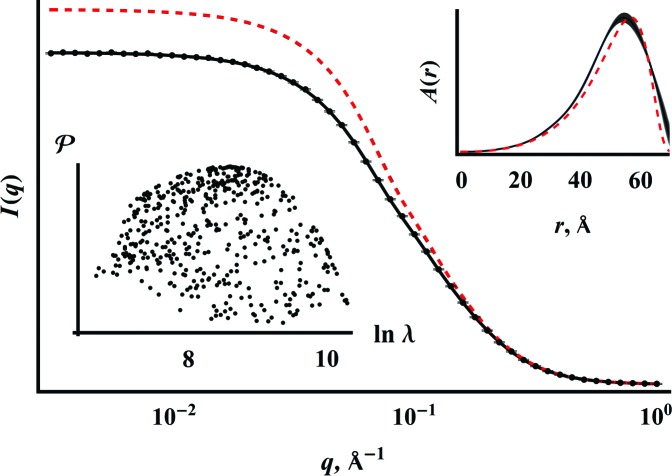
Simulated 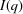 data with 1% added Gaussian noise before (dashed red) and after smearing (points); the solid line shows the fit 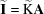. The desmeared data come from the solution 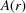 *via* 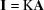, where the pinhole-collimated operator 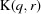 results from substitution of a delta function for the slit geometry 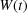 in equation (6). The upper inset shows the generating distribution (dashed red) and the reconstruction 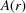 (black); the lower inset shows a projection of the evidence 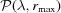 surface onto the 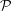
–λ plane during the search for 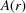.

Figs. 1 ▸ and 2 ▸ illustrate the power of Bayesian–MaxEnt methods as implemented in Hansen’s *IFTc* (Hansen, 2014 ▸) applied to SAS data analysis using a set of simulated scattering data from an LSW ensemble of spheres according to equation (5): 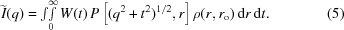
This formula assumes isotropic scattering from a line-collimated incident beam with the slit orthogonal to the direction of resolution. Integration of a spherical form factor over the distribution 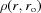 and the slit geometry 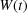 gives the slit-smeared scattered intensity 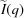. The simulated data result from addition of 1 and 10% random Gaussian error, respectively.

Reconstruction of the distribution 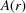 uses the inverse problem formulation in equation (6):
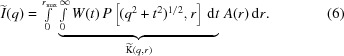
Integration of the spherical form factor 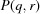 over the slit geometry 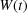 gives the transform matrix elements 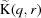, and discretization gives the matrix equation 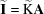. The matrix equation accommodates a flat background in 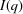 by appending an element to 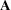 and a column of ones to the operator 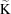. Bayesian methods can be used to find a solution to equation (6) by maximizing the evidence 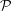 for the solution. Laplace’s approximation of the posterior,
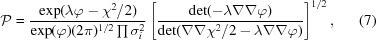
gives 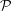 as a function of the hyperparameters Lagrange multiplier λ and limit of integration 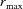 (Hansen, 2000 ▸; Vestergaard & Hansen, 2006 ▸). The measurement error σ, together with the prior and goodness of fit, uniquely determines the most likely value for the evidence.

A small simulated measurement uncertainty (Fig. 1 ▸) gives a MaxEnt reconstruction of 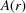 that closely follows the originating LSW distribution. Addition of 10% random noise causes the reconstructed 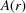 to differ significantly from the original input data. Less convincing data means that the result cannot differ as significantly from the prior expectation, so a simpler (in this context, closer to the flat prior expectation) distribution than the original LSW results. The reason for the drastic change in the appearance of 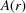 with these relatively small amounts of added error in 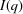 becomes clear by comparing the similarity between the desmeared scattering intensity of the original (error-free) data and the reconstruction shown by the black and red dashed lines in Fig. 1 ▸. These examples serve to illustrate the intuitive nature of the Bayesian–MaxEnt approach – the quality of the measurement influences the information obtainable from the data analysis.

Characterization techniques often employ SAS data alone for determination of the PSD owing to its ease of use *in situ*; in these situations, indiscriminate use of a prescribed size distribution risks biasing the conclusion toward the preconceived result. The appeal of MaxEnt comes from the inherent ability to extract as much information from SAS data as possible without overfitting. However, application of MaxEnt to metallic alloys becomes complicated by two effects: the grain structure of metals typically gives power law scattering at low *q* and interparticle interference [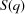] appears in relatively high volume fraction alloys. Conventional inverse transformation using equation (2) gives misleading results in the absence of flat 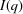 at low *q* (the distribution relates to the *q* range of the instrument, rather than the underlying sample) and can fail with significant 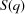 influence. In this work, we extend the inverse transformation methodology to accommodate both effects, and demonstrate its utility by analyzing temporal *in situ* SAXS from precipitation hardening in a simple nickel-based alloy system.

## 2. Experimental   

Ni–Al–Si alloys were prepared from the pure metals in an arc-melting furnace with a copper hearth, with subsequent homogenization at 1373 K. The specimen was electrical discharge machined, wet polished to a thickness of 60 µm and placed in the cup of a Linkam 1500 furnace. The furnace was placed in the USAXS–SAXS–WAXS instrument and heated to an indicated temperature of 873 K, and USAXS–SAXS–WAXS scans were taken over a period of 200 min. The X-ray energy was 24 keV, the exposure times for SAXS and WAXS were 30 s, and the USAXS flyscan time was 90 s.

**Figure 3:**
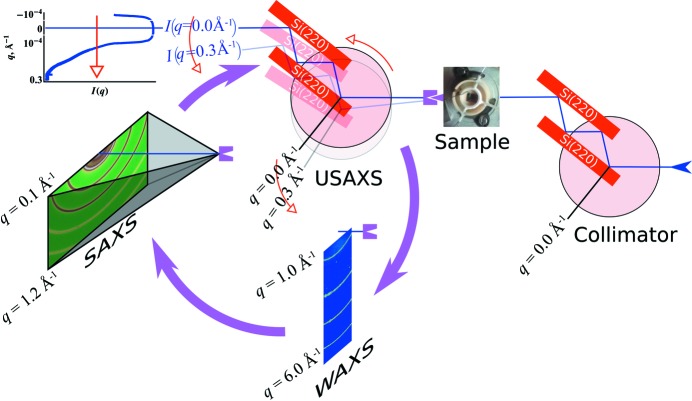
Schematic of the USAXS–SAXS–WAXS instrument. The pair of collimating crystals and the sample remain fixed, while the USAXS, SAXS and WAXS components move in and out around the sample. The Bonse–Hart USAXS analyzer stage rotates and moves down while scanning in *q*, while SAXS and WAXS data come from conventional area detectors.

The USAXS–SAXS–WAXS instrument (Ilavsky *et al.*, 2009 ▸, 2013 ▸; Fig. 3 ▸) consists of a Bonse–Hart USAXS instrument combined with two-dimensional SAXS and WAXS area detectors. The first pair of Si(220) optics (‘collimator’) removes significant higher harmonics from the upstream Si(111) monochromator and remains in place for all three modes of operation. In the USAXS mode, a second pair of Si(220) crystals (‘USAXS’) is moved in front of the sample and scans the USAXS *q* range that includes the main beam from 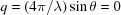 to 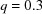
 Å^−1^ with a resolution of 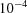
 Å^−1^ in the scanning direction and 0.3 Å^−1^ orthogonal to the plane of the analyzer crystals. SAXS and WAXS employ two-dimensional area detectors with *q* ranges from 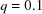
 Å^−1^ to 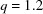
 Å^−1^ and 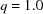
 Å^−1^ to 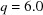 Å^−1^, respectively. Effective slit smearing of the USAXS data in the direction perpendicular to the scanning direction means that combining the USAXS with the SAXS data requires mathematical slit smearing of the pinhole-collimated SAXS. Altogether, the USAXS–SAXS–WAXS instrument covers a range from 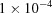 to 6 Å^−1^ (corresponding to length scales from 6 µm to 1 Å) with a typical time resolution of 5 min.

## 3. Results and discussion   

**Figure 4:**
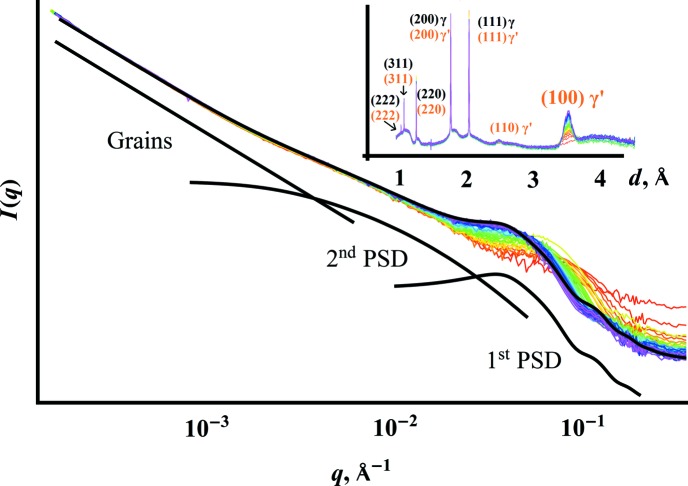
USAXS–SAXS–WAXS during *in situ* annealing of a ternary Ni alloy. The combined USAXS–SAXS data show structural alloy evolution beginning from the homogenized material, exhibiting a flat high-*q* region (red) progressing to formation and growth of precipitates. The inset shows the corresponding WAXS, revealing the change in phase composition. The 100 reflection grows with the shift in the high-*q* population, consistent with growth of the 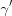 phase. The black line shows one example of the hierarchical model used to fit the data over time. The lines underneath show the linearly superposed fitting components of the grain structure, the time-invariant PSD and the dynamic PSD corresponding to the growing precipitates.

*In situ* time-resolved USAXS–SAXS–WAXS on an Ni–Al–Si alloy undergoing heat treatment at 873 K yielded the time-resolved data series shown in Fig. 4 ▸. The initial scattering intensity 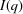 shows a low-*q* power law slope and a Guinier knee at mid-*q*. During heat treatment, a new population of scatterers appears at high *q* and shifts to lower *q* with time, as expected from previous studies of precipitation behavior in this alloy system (Muralidharan & Chen, 2000 ▸). The combined USAXS–SAXS data after heat treatment suggest the presence of three length scale regimes, namely a low-*q* power law region for the grain structure of the metal and two precipitate size distributions:
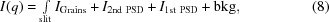


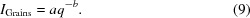
The following analysis of precipitate evolution during heating ignores the invariant second PSD, fitting it with a normally distributed 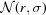 ensemble of spheres:
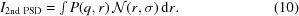
The high-*q* region shows growth of a new first PSD, consistent with precipitation hardening in Ni alloys. At the same time, the WAXS data reveal the gradual appearance of the 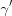 phase during heating, distinguishable by its 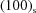 superlattice reflection.

Studies of similar alloys using transmission electron microscopy revealed spherical precipitates on the smallest length scales (Muralidharan & Chen, 2000 ▸), suggesting use of a spherical form factor integrated over a distribution for the first PSD. Preliminary analysis revealed that 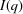 in this region fell more sharply than Guinier’s law, suggesting use of an interparticle interference function 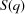 in conjunction with the single-particle scattering using the approximation 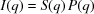. Equation (11) shows the resulting structural level for the first PSD, 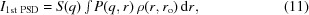
where 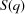 indicates the interprecipitate structure factor and 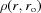 the LSW distribution. The scripting tool in the software package *Irena* (Ilavsky & Jemian, 2009 ▸) was used to fit all the measured data sequentially to this hierarchical USAXS–SAXS model; the 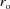 thus obtained follows the 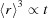 time dependency predicted by LSW theory. However, some assumptions of LSW theory, such as a dilute system of precipitates, may not apply to real systems. MaxEnt techniques remove an initial bias by avoidance of an *a priori* assumption about the form of the distribution.

The presence of the grain structure, second PSD and structure factor together makes implementation of MaxEnt difficult; a variety of existing modifications address inverse transformation of SAXS data in the presence of 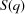. In cases with weak correlation between particles, simple truncation of the data at low *q* may serve to isolate a sufficient range of uncontaminated 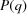. For spheroidal particles at moderate volume fractions, an extension to *IFTc* elucidates estimates for 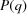 and 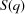 (Hansen, 2008 ▸). The generalized indirect Fourier transform (GIFT) method accommodates interparticle interactions by fitting an effective 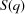 model and performing the inverse transformation (Brunner-Popela & Glatter, 1997 ▸). This method fixes the Lagrange multiplier while searching for 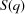 parameters based upon the misfit 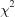. Here, we propose integration of the GIFT technique with the Bayesian solution for 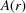. In addition to treatment of the Lagrange multiplier as an unknown hyperparameter in the original Bayesian implementation of MaxEnt, our approach also optimizes the 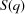 (hyper)parameters using the evidence obtained for the resulting PSD 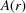.

For study of this alloy system with MaxEnt, the form of the inverse problem changes from its typical implementation [equation (3)] to include a product with a structure factor 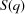:
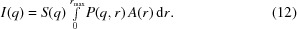
Because 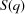 and its parameters do not depend on *r*, the inverse problem becomes
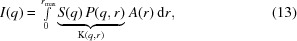
again in the form of a matrix equation 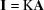. The corresponding inverse problem 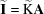 for line-collimated data comes from integration of the kernel over the slit geometry:
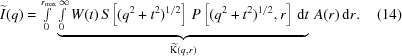



The conventional analysis in Fig. 4 ▸ used a power law slope for the grain structure and a static collection of spheres for the low-*q* PSD. Because the grain scattering and low-*q* PSD evidently do not change over the course of this study, truncation of the data in the region near 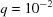
 Å^−1^ gives a single power law slope at low *q* that results from the terminal slope of the second PSD. Defining an effective structure factor 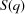 as resulting from the low-*q* power law and an interference function for hard spheres given by Vrij (1978 ▸) gives the following model:
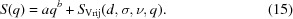
The effective structure factor 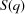 has parameters mean distance *d*, variance σ and volume fraction ν characterizing hard sphere interaction, and *a* and *b* characterize the intensity and terminal slope of the invariant secondary PSD. While this method requires an initial assumption about 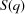, Fritz & Glatter (2006 ▸) determined that the solution of inverse problems such as these does not depend heavily on this choice, to the extent that averaging the diagonal elements of the partial structure factor matrix for hard spheres gives acceptable results. The classic MaxEnt problem sought a Lagrange multiplier λ that gave the most likely solution 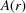. Here, we seek the values of λ, *d*, σ, ν, *a* and *b* that give the most likely solution 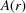.

Unlike the λ and 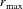 hyperparameters in the classic small-angle MaxEnt kernel, the nonlinearity of 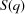 hyperparameters does not guarantee a single maximum in the multidimensional evidence posterior, precluding use of the Laplace approximation. In addition, Monte Carlo approximation of the posterior would require recalculation of the transform matrix elements, including the internal partial structure factor parameters, for each point. The alternative used herein treats the problem within a hierarchical Bayesian framework.

Fixed values for the structure factor hyperparameters and performing MaxEnt according to the model in equation (14) gives a solution 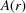 with its most likely value for the hyperparameters λ. Unlike MaxEnt without an 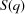, computational efficiency dictates a fixed value for 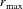, since changing it requires recomputing the transform elements. The solution 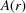 obtained from a particular set of structure factor hyperparameters has a value for the associated evidence 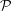. The best solution for 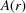 comes from finding parameters for the structure factor that give the greatest evidence 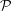. The search for structure factors at this outer level of inference uses Goffe’s implementation of Monte Carlo Metropolis–Boltzmann annealing (Goffe *et al.*, 1994 ▸). A broad predefined in­ter­val for the 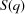 parameters corresponds to an uninformative flat prior. Boltzmann annealing narrows this interval and focuses on a region of the parameter space that has the greatest evidence. Changes in 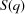 eventually become small enough that 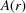 and 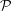 no longer significantly change, ending the optimization.

**Figure 5:**
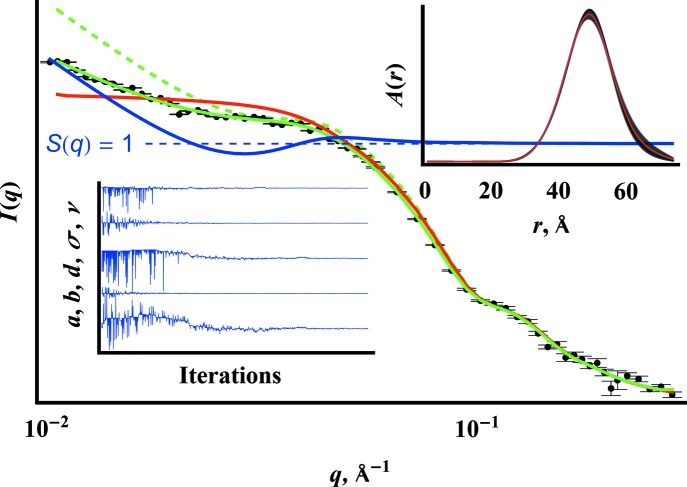
Application of the model in equation (14) to a data set (black points) from Fig. 4 ▸, showing the smeared fit found (solid green), the desmeared equivalent (dashed green), the effective structure factor as defined in equation (15) (blue) and the scattering intensity from a noninteracting ensemble of spheres (red). The upper inset shows the distribution 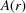 obtained; the black band shows the error approximated using the posterior evidence distribution. The lower inset shows the progression of the Boltzmann–Metropolis search for the best 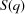 parameters.

**Figure 6:**
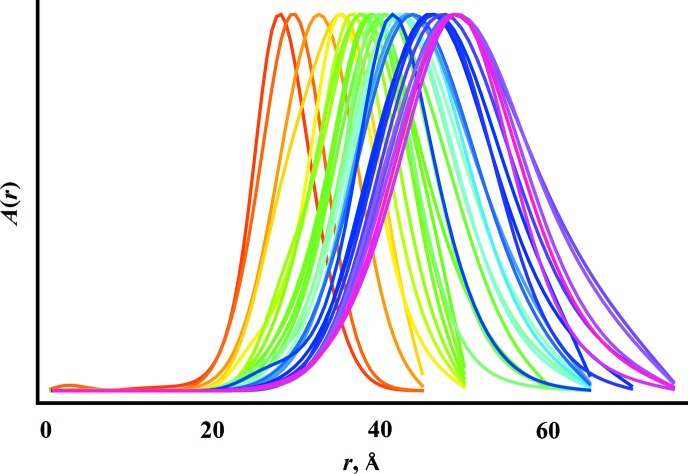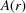 obtained using the model in equation (14) from the temporal USAXS–SAXS data shown in Fig. 4 ▸, from the initial appearance of the precipitate (red) to its PSD after heating at 873 K for 3.5 h (magenta).

Fig. 5 ▸ demonstrates this technique applied to one 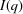 from the temporal USAXS–SAXS shown in Fig. 4 ▸. Truncation of the data removes the low-*q* scattering from the grain boundaries and the secondary particle size distribution, leaving a power law slope at low *q*. As shown by the plot parameter values as a function of iteration number, the search algorithm begins with a random search over a wide range of values, searching for a region with a global maximum. At the end, the values for 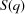 hyperparameters having the highest evidence give the most likely 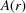 consistent with the data, measurement error and assumption about the form of 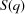. Analysis of the data set shown required about 30 min over eight threads. After setting the flat priors for 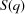 and fixing 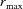, the program automatically finds the solution 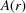. Fig. 6 ▸ shows the series of results for 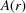 resulting from the temporal USAXS–SAXS data in Fig. 4 ▸.

**Figure 7:**
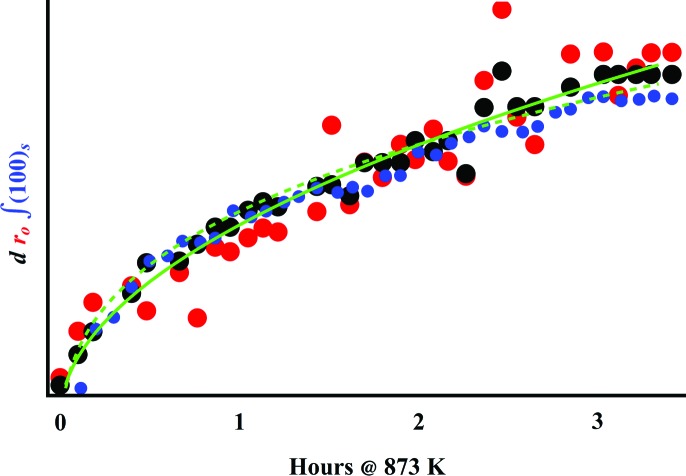
Comparison of parameters obtained from the mode 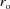 of each 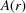 (red), the *d* values simultaneously obtained from the 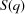 model (black) and the integrated intensity of the 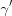 superlattice reflection 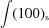 (blue). For comparison, the green lines show the best fit to 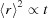 (solid) and 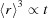 (dashed).

As a check of the technique’s analytical methodology and internal consistency, in Fig. 7 ▸ we compare the particle size determined from 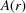, the particle size determined from the Bayesian search for the 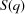 hyperparameters and the kinetics revealed by WAXS. Defining 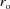 as the mode of 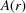 gives a method for characterizing the spherical precipitate sizes present. The most likely 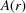 comes from the most likely value for the 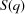 hyperparameters, which includes the diameter *d* of spheres in Vrij’s structure factor 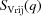. The WAXS shows growth with time of the 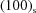 superlattice reflection, characterized by its integrated intensity 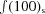. Plotting these three parameters on the same time axis demonstrates that all three follow similar trajectories. At longer times, the evolution approaches 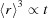.

## 4. Conclusion   

Small-angle scattering allows *in situ* volume-averaged measurements on alloys during heat treatment. Analysis of the scattering data using conventional methods relies on external prior knowledge about the form and distribution of precipitates. The desire to avoid prior assumptions about the form of the PSD solution drove the development of MaxEnt approaches that seek the best explanation for the PSD given only the measured data and the shape of the precipitates. However, alloys that do not have flat 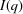 at low *q* or have 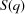 present generally inhibit application of the MaxEnt method. We have described a Bayesian augmentation of the GIFT technique for MaxEnt determination of PSDs from scattering of such alloys. Its application to *in situ* USAXS–SAXS–WAXS data collected from a model Ni–Al–Si system revealed a self-consistent correlation between the result of the distribution, the 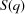 parameters and the phase identity from WAXS.

The Fortran program employed in this work uses a slightly modified version of *BayesApp*’s *IFTc* (Hansen, 2012 ▸, 2014 ▸) as an internal subroutine. The correspondence author will provide the source code and a Windows executable for free upon request.

## Figures and Tables

**Figure 2:**
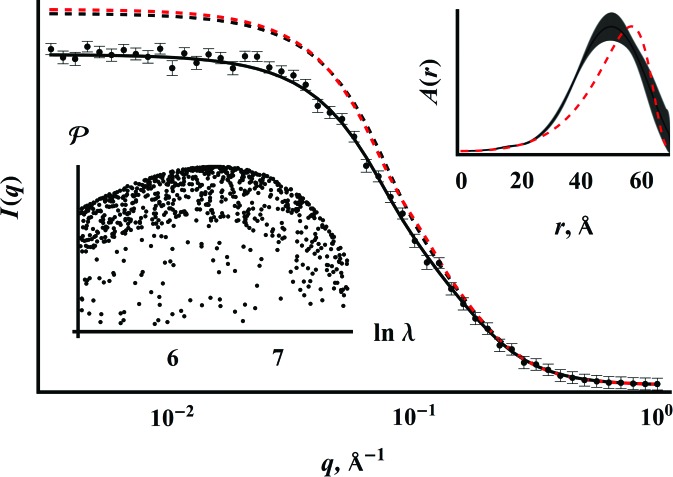
The same data and analysis technique presented in Fig. 1 ▸, but with 10% added Gaussian error. The width of the reconstruction 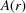 line (black) reflects the error estimate.
